# Diagnostic Yield of Repeat Endoscopic Ultrasound-Guided Fine Needle Biopsy for Solid Pancreatic Lesions

**DOI:** 10.3390/cancers15143745

**Published:** 2023-07-24

**Authors:** Baptiste Camus, Anna Pellat, Alexandre Rouquette, Ugo Marchese, Anthony Dohan, Arthur Belle, Einas Abou Ali, Stanislas Chaussade, Romain Coriat, Maximilien Barret

**Affiliations:** 1 Department of Gastroenterology and Digestive Oncology, Cochin Hospital, Assistance Publique-Hôpitaux de Paris, 75014 Paris, France; 2 Unité de Formation et de Recherche de Médecine, Université Paris Cité, 75006 Paris, France; 3 Department of Pathology, Cochin Hospital, Assistance Publique-Hôpitaux de Paris, 75014 Paris, France; 4 Department of Digestive Surgery, Cochin Hospital, Assistance Publique-Hôpitaux de Paris, 75014 Paris, France; 5 Department of Abdominal and Interventional Imaging, Cochin Hospital, Assistance Publique-Hôpitaux de Paris, 75014 Paris, France

**Keywords:** EUS FNB, EUS FNA, pancreatic neoplasm, pancreatic ductal adenocarcinoma

## Abstract

**Simple Summary:**

Histological sampling is the cornerstone of diagnosis for patients with solid pancreatic lesions. Endoscopic ultrasound guided fine needle biopsy (EUS FNB) is the most commonly used pancreatic tissue sampling technique. Our aim was to assess the diagnostic yield of a second EUS FNB after a first negative one. The second puncture enabled histological diagnosis in 74% of cases, making it a very efficient procedure in this situation according to this study. In addition, morbidity was very low. The rate of benign diagnoses was 26% after a negative first puncture, which tends to suggest a second puncture rather than surgery in this situation.

**Abstract:**

Patients and methods: we performed a retrospective case-control study, including cases with repeat EUS FNB for a solid pancreatic lesion, matched on a 1:2 ratio on age, sex, tumor location and presence of chronic pancreatitis with cases diagnosed on the first EUS FNB. Results: thirty-four cases and 68 controls were included in the analysis. Diagnostic accuracies were 80% and 88% in the repeat and single EUS FNB groups, respectively (*p* = 0.824). The second EUS FNB had a sensitivity of 80%, a specificity of 75%, a positive predictive value of 96%, and a negative predictive value of 33%. Of the 34 patients in the repeat EUS FNB group, 25 (74%) had a positive diagnosis with the second EUS FNB, 4 (12%) after surgery due to a second negative EUS FNB, 4 (12%) during clinical follow-up, and 1 (3%) after a third EUS FNB. Of the 25 patients diagnosed on the repeat EUS FNB, 17 (68%) had pancreatic adenocarcinomas, 2 (8%) neuroendocrine tumors, 2 (8%) other autoimmune pancreatitis, 2 (8%) chronic pancreatitis nodules, 1 (4%) renal cancer metastasis, and 1 (4%) other malignant diagnostic. There were no complications reported after the second EUS FNB in this study. Conclusion: repeat EUS FNB made a diagnosis in three fourths of patients with solid pancreatic lesions and a first negative EUS FNB, with 26% of benign lesions. This supports the repetition of EUS FNB sampling in this clinical situation.

## 1. Introduction

Endoscopic ultrasound (EUS)-guided tissue sampling is the recommended diagnostic modality for solid pancreatic lesions, mainly represented by pancreatic ductal adenocarcinoma [1,2,3]. The diagnostic performances of EUS-guided fine needle aspiration (FNA) for solid pancreatic lesions are respectively 83%, 100%, 100%, 72%, and 88% for sensitivity, specificity, positive predictive value, negative predictive value, and diagnostic accuracy [4]. Previous findings have shown that the diagnostic accuracy of a second EUS FNA is 61–84% [5], reaching an overall of 83–96% with two procedures [6].

During the last decade, however, EUS FNA has been replaced by EUS-guided fined needle biopsy (EUS FNB), which gave access to core tissue samples for histological analysis and improved the diagnostic accuracy with less needle passes [7]. In the meantime, the EUS-guided sampling technique has been standardized, and the use of the stylet slow-pull technique widely adopted [8]. In case of an inconclusive EUS FNA or FNB, the course of action is not clearly defined by the European Society of Digestive Endoscopy guidelines [9]. Review of the pathology slides by another pathologist, upfront surgical resection, or a second EUS guided sampling can all be considered [1]. Several works have been conducted to assess the value of a repeat EUS FNA [5,6], but none with FNB.

The aim of this study was to determine the diagnostic yield of a second EUS FNB after a first inconclusive one.

## 2. Patients and Methods

**Figure 1:**
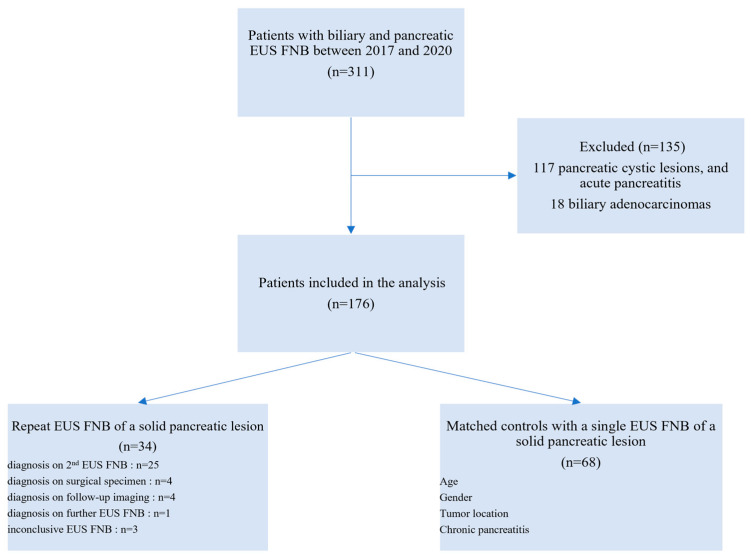
Flow chart of the study participants. EUS: Endoscopic Ultrasound. FNB: Fine Needle Biopsy; IPMN: Intraductal Papillary Mucinous Neoplasms.

The primary endpoint was the diagnostic accuracy of repeat EUS FNB, defined as the ratio of the sum of true positives and true negatives to the total number of examinations. The secondary endpoints were the diagnostic performances of the repeat EUS FNB after a first negative one, and the safety of this strategy. We included all consecutive patients who underwent at least two EUS FNB for solid pancreatic lesion between January 2017 and November 2020. Patients were selected from a prospectively collected database listing all patients with EUS-guided tissue sampling at our institution. Patients who had a single EUS FNB for pancreatic mass over the same time interval were used as controls. Patients with cystic pancreatic lesions, biliary tract tumors (cholangiocarcinoma and carcinoma of the ampulla), and extra-pancreatic solid lesions tumors were excluded from the analysis. The study flowchart is presented in Figure 1.

For each patient and each EUS FNB, we retrospectively collected demographic data, patients’ medical history, procedural characteristics of the EUS FNB, EUS findings, histological results, complications (according to the ASGE Lexicon [10]), and the modality of the final diagnosis (first, second or subsequent EUS FNB, surgery or evolution).

The study group included patients with two or more EUS FNB for the diagnosis of solid pancreatic lesions. These patients were compared with a control group of patients who had a diagnosis of solid pancreatic lesions obtained on the first EUS FNB. The patients were matched on a 1:2 ratio, on the following characteristics: age, sex, tumor location, and presence of chronic pancreatitis. Baseline patient characteristics are presented in Table 1.

**Figure 2:**
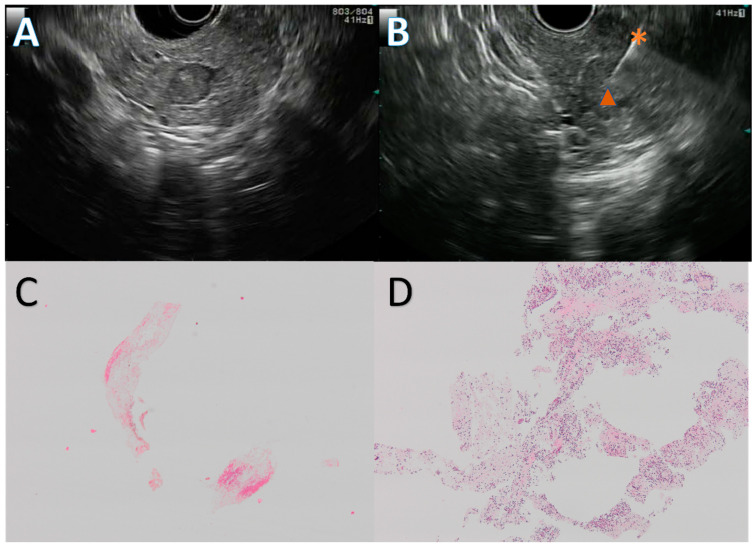
Endoscopic ultrasound guided fine needle biopsy (EUS FNB) of a pancreatic neuroendocrine tumor. Solid, 10 mm, hypoechogenic round lesion in the pancreatic head (**A**) first punctured with a 20 G Procore needle with inconclusive results, then with a 22 G Acquire needle (**B**) during a repeat EUS FNB. The first EUS FNB showed only fibrin and red blood cells (**C**), while the second one retrieved multiple neuroendocrine-like cells (**D**). Figure (**C**,**D**) are at ×4 magnification. The diagnosis was confirmed by a strong anti-synaptophysin antibody staining on immunohistochemistry. *: 22 G Acquire needle, ▲: neuroendocrine tumor.

The EUS procedures were carried out in patients in left lateral position, under deep sedation, by three experienced endosonographists, using a linear array echoendoscope (GFUCT-140, 160 or 180, Olympus, Tokyo, Japan) and an FNB needle. The choice of the needle, between 20 G Procore, 22 G Procore (Cook Medical, Bloomington, IN, USA), or 22 G Acquire (Boston Scientific, Natick, MA, USA) was left to the discretion of the endosonographist. The stylet slow-pull technique was used in all instances, and two to three passes were performed, depending on the macroscopic on-site evaluation. The samples were collected and fixed in 10% formalin for 12–24 h, and then centrifuged. The specimen was embedded in paraffin, cut into 4 μm section, and stained with HES. A representative EUS FNB procedure and the corresponding histological slides are presented in Figure 2.

Patients’ characteristics are presented as mean ± standard deviation (SD). We used *t*-test to assess the differences between the groups for numerical variables and a chi-square test for categorical variables. We considered *p* value < 0.05 to indicate statistical significance. Statistical analyses were performed using RStudio Software version 2022.07.0 (R Foundation for Statistical Computing, Vienna, Austria).

Our study received approval from our local institutional review board (CLEP number AAA-2022-08051).

## 3. Results

Of the 34 patients in the repeat FNB group, 25 (74%) had a diagnosis with the second EUS FNB.

Operating characteristics: there was no difference in diagnostic accuracy between the single EUS FNB group (88%) and the repeat EUS FNB group (80%) (*p* = 0.824). The second EUS FNB had a sensitivity of 80%, a specificity of 75%, a positive predictive value (PPV) of 96%, a negative predictive value (NPV) of 33%, and a diagnostic accuracy of 80%. These results are presented in Table 2.

In the repeat EUS FNB group, the first negative puncture was classified into 3 categories: negative, meaning normal pancreas (53%, 18 patients); inconclusive, meaning abnormal pancreatic tissue but without suspicious cells (38%, 13 patients); and inadequate, meaning non-contributory sampling (9%, 3 patients).

Among patients with a negative second EUS FNB, four (12%) were diagnosed after surgery, four (12%) during clinical and radiological follow-up and one (3%) after a third EUS FNB. Twenty (59%) patients had a diagnosis of pancreatic adenocarcinoma, three (9%) of neuroendocrine tumor, five (15%) of chronic pancreatitis, three (9%) of autoimmune pancreatitis and one (3%) of pancreatic metastasis. Other diagnosed lesions were degenerated IPMN (one (3%) patient), and normal pancreas (one (3%) patient). These results are presented in Table 3.

Of the 25 patients diagnosed on the second EUS FNB, 17/25 (68%) had pancreatic adenocarcinomas, 2/25 (8%) neuroendocrine tumors, 2/25 (8%) autoimmune pancreatitis, 2/25 (8%) chronic pancreatitis, 1/25 (4%) renal cancer metastasis and 1/25 (4%) other malignant diagnostic (degenerated IPMN). Of the four patients diagnosed by surgery after failure of two or more EUS FNBs, one patient had a diagnosis of neuroendocrine tumor, one of pancreatic adenocarcinoma, and two of chronic pancreatitis nodule with no malignancy. Among the four patients diagnosed during clinical follow-up, three patients had a benign diagnosis (autoimmune pancreatitis, chronic pancreatitis nodule and normal pancreas), and one patient probably had a pancreatic adenocarcinoma suggested by the rapid evolution of symptoms without final histological documentation. One patient had a diagnosis of pancreatic adenocarcinoma after a third EUS FNB.

The proportion of benign diagnoses was 26% (9/34 patients) in the repeat EUS FNB group, and seems to be higher than in the single EUS FNB group (6%, 4/68 patients).

In our center, four types of needles compatible with the FNB technique are used: 22 G Procore; 20 G Procore; 25 G Procore; and 22 G Acquire. Among the patients punctured once (single EUS FNB group), the 20 G Procore was the most commonly used needle, with 49 punctures (74%), followed by the 22 G Acquire (6 punctures, 9%), the 22 G Procore (6 punctures, 9%), and the 25 G Procore (2 punctures, 3%). Among patients punctured twice, the needle used for the first puncture was the 20 G Procore (17 punctures, 50%), followed by the 22 G Procore (11 punctures, 32%), 22 G Acquire (4 punctures, 12%) and 25 G Acquire (2 punctures, 6%). In the same group, the needles used for the second puncture were the 20 G Procore (68%, 23 punctures), the 22 G Acquire (15%, 5 punctures), the 22 G Procore, and 25 G Procore (9% each, 3 punctures for each needle). The use of 22 G Acquire and 20 G Procore needles was more frequent during the second puncture than during the first (15 vs. 12% and 68 vs. 50%, respectively). The 22 G Procore needle was overrepresented in the repeat EUS FNB group (32% vs. 9%, *p* = 0.008). Conversely, there was a greater use of the 20 G Procore needle in the single EUS FNB group compared with the repeat EUS FNB group (74% vs. 50%, *p* = 0.028). Among patients punctured twice, the needle used for the second puncture was mostly the 20 G Procore (24 punctures, 71%), followed by the 22 G Acquire (5 punctures, 15%), 22 G Procore (3 punctures, 9%), and 25 G Acquire (3 punctures, 9%).

In this study, the mean number of needle passes was 2.36 in the single EUS FNB group versus 2.29 in the repeat EUS FNB group (*p* = 0.617) at the first puncture. The mean needle passes between the first and second puncture in the repeat EUS FNB group were 2.56 vs. 2.29, *p* = 0.134.

Complications were recorded in 7 (11%) and 3 (9%) patients in the single and repeat EUS FNB group, respectively. For the double EUS FNB group, all complications were after the first puncture; there were no complication reported after the second puncture in this study. Complications were mainly bleeding (three patients, 43%) in the single EUS FNB group, and mainly pancreatitis (two patients, 67%) in the repeat EUS FNB group. There was no observable difference in terms of severity of complication, with one case in each group of severe complication without death.

## 4. Discussion

In our work, we observed a 74% rate of confirmed histological diagnosis on a repeat EUS FNB for solid pancreatic lesions, including a 26% rate of benign diagnoses. There was a significant difference in benign diagnoses between the single and double EUS FNB group in favor of the double EUS FNB group, supporting the possibility of a greater proportion of benign diagnoses after a first negative EUS FNB. There were no significant adverse events. Following two EUS FNB negative for malignancy, the final diagnosis was benign in half of cases.

It is important to underline that current guidelines do not require histopathology before surgical resection of a pancreatic mass that is resectable and suggests pancreatic adenocarcinoma. However, the majority of pancreatic lesions do not meet these criteria, and actually require histological documentation. Percutaneous sampling of the pancreas is of course possible, but requires great expertise from the interventional radiologist to avoid biopsying through the digestive tract. Finally, tumor seeding on the puncture tract, although possible in theory, does not seem to be a significant clinical problem, as confirmed by various works [11,12].

EUS FNB is a very efficient technique for the diagnosis of pancreatic solid tumors. In an Italian cohort of 463 patients punctured and operated, its diagnostic performances showed 100% sensitivity, 93% specificity, 97% PPV, 100% NPV and 93% of diagnostic accuracy. Moreover, no severe complications were recorded in this work [13]. These results are consistent with a 2016 meta-analysis, including 16 articles and 828 patients, which found similar diagnostic performances (84% sensitivity, 98% specificity, 100% PPV, 17% NPV, and 96% diagnostic accuracy) [14]. Of note, these diagnostic performances partly depend on the type of needle used: a 2020 study compared the performance of the 20 G Procore FNB needle to that of the 22 G Acquire FNB needle, and concluded to the superiority of the latter in terms of diagnostic accuracy (100% vs. 87%, *p* = 0.001) and quality of the biopsy specimen (82% vs. 67%, *p* = 0.02) [15].

There is no consensus on the management after a non-conclusive first EUS FNB. A second EUS FNB is only one of the possible options, but to our knowledge, the diagnostic yield of the repeat EUS FNB has never been evaluated in the setting of suspected malignancy. On the contrary, the value of a repeat EUS FNA has been well evaluated. In a 2016 study, including 45 repeat EUS FNA of which 34 had a non-conclusive histology at the first puncture, Mitchell and al. found a conclusive diagnosis after a repeat EUS FNA in 59% of cases (20/34 patients) [16]. However, this work only evaluated FNA needles. Similarly, in 2008, Tadic et al. published a series of 46 EUS FNA, of which 9 were non-conclusive. After a repeat EUS FNA, 78% of patients (8/9) had a diagnosis of malignancy. For the first EUS FNA, sensitivity, specificity, positive predictive value, negative predictive value and diagnostic accuracy were 68%, 100%, 100%, 73%, and 83%, respectively. For the second EUS FNA, sensitivity, NPV and diagnostic accuracy were 92%, 77%, and 96%, respectively. However, this work was based on a small number of patients, and only FNA needles were used [6]. In a 2020 meta-analysis including 505 patients, there was a 77% sensitivity, a 98% specificity, a 99% PPV, and a 61% NPV for repeat EUS FNA. However, these diagnostic performances were dependent on the presence of rapid on-site evaluation (ROSE) of the tissue sample [17]. EUS FNB has the advantage of providing the same diagnostic performance regardless of the presence of ROSE [18].

In recent series of patients operated for pancreatic tumors without prior histological diagnosis, the probability of operating a patient for a benign diagnosis was 7% [19]. In our study, the proportion of patients with a benign diagnosis after a second conclusive EUS FNB procedure was 16% (4/25 patients). Among patients with a non-conclusive second EUS FNB (9/34 patients), 5 patients finally had a benign diagnosis (2 after surgery, 3 on the clinical and radiological follow-up). A total of 26% (9/34) of patients finally had a benign diagnosis when the first EUS FNB was negative. This suggests that the risk of surgically resecting a benign pancreatic lesion is greater in the population of patients with a non-contributive first EUS FNB.

The second generation of FNB needles (e.g., 22 G Acquire needle, Boston Scientific, Marlborough, MA, USA) has allowed a diagnostic accuracy of 87% [15]. The mismatch with the results of our study can be explained by the small proportion (11%) of 22 G Acquire needles used in our center during the study period. 

Our results support the repetition of EUS FNB in the case of a first negative EUS FNB for a solid pancreatic lesion. Indeed, this attitude led to a diagnosis in three-fourths of patients. In case of a negative second EUS FNB, considering an additional diagnostic yield of 11% (1/9 patient) for a third EUS FNB, it seems preferable to opt for a close clinical and radiological follow-up. The challenge is not to miss the diagnosis of malignant tumor, which remain possible in about 50% of cases in this study, in order not to worsen the prognosis by therapeutic delay. Considering the complication rate of pancreatic resections, we believe that surgery without histological documentation should be avoided, or at least discussed on a case-by-case basis in a multidisciplinary meeting.

The main limitation of the study is its retrospective nature, although data collection was done prospectively, and allowed for the inclusion of consecutive patients. Matching was used to limit bias by increasing the comparability of patients included. Matching on the location and presence of a chronic pancreatitis seemed fundamental. Indeed, the technical difficulties associated with these two characteristics can result in altered sample quality and increase the difficulty of interpretation by the pathologist. The statistically significant difference in tumor size between the two groups, with larger tumors observed in the single EUS FNB group (25+/−9 mm vs. 31+/−13 mm, *p* = 0.010), is a limitation of our work. Indeed, the greater technical difficulty of puncturing a small lesion leads to a greater probability of having an inconclusive result and a repeat EUS FNB. Most patients of the double EUS FNB group were punctured twice in our center (68%), of which 21% were punctured by two different operators. Nevertheless, practices are standardized, which limits the bias related to the puncture technique. There was a significant difference in tumor size between the two groups with larger tumors observed in the single EUS FNB group (25+/−9 mm vs. 31+/−13 mm, *p* = 0.010). The greater technical difficulty of puncturing a small lesion leads to a greater probability of having an inconclusive result and a repeat EUS FNB. Finally, the inclusion of the patients in a single center—although several endoscopists were involved—might limit the generalizability of our results. 

In this study, a second EUS FNB led to a diagnosis in 3 cases out of 4, with few complications. Twenty-six percent of patients finally had a diagnosis of a benign lesion (autoimmune pancreatitis or chronic pancreatitis nodule). In our eyes, these results would support the repetition of EUS FNB rather than upfront surgical resection in the case of a first negative EUS FNB.

## 5. Conclusions

Repeat EUS FNB made a diagnosis in three-fourths of patients with solid pancreatic lesions and a first negative EUS FNB, with 26% of benign lesions. This supports the repetition of EUS FNB sampling in this clinical situation.

## Figures and Tables

**Table 1:** Baseline characteristics of the study cohort.

	Repeat EUS FNB Group *n* = 34	Single EUS FNB Group *n* = 68	*p* Value
Age at diagnosis (mean (SD))	68 (10.38)	69 (10.14)	0.759
Gender			0.723
Female—*n* (%)	13 (38)	30 (44)	
Male—*n* (%)	21 (62)	38 (56)	
Tumor location			0.496
Head—*n* (%)	22 (65)	40 (59)	
Body—*n* (%)	5 (15)	10 (15)	
Tail—*n* (%)	2 (6)	11 (16)	
Uncinate process—*n* (%)	5 (15)	7 (10)	
Tumor size, in mm—mean ± SD	25 ± 9	31 ± 13	0.010
Presentation			
Jaundice—*n* (%)	10 (29)	27 (41)	0.295
Abdominal pain—*n* (%)	8 (23)	12 (18)	0.363
Weight loss—*n* (%)	3 (9)	6 (9)	0.712
Pancreatitis—*n* (%)	1 (3)	6 (9)	1.00
Diabetes—*n* (%)	1 (3)	3 (5)	0.467
Abnormal liver tests—*n* (%)	2 (6)	0 (0,0)	1.00
Digestive obstruction—*n* (%)	1 (3)	1 (2)	0.216
Incidental finding on abdominal imaging—*n* (%)	5 (15)	10 (15)	1.00
Other—*n* (%)	3 (9)	1 (2)	0.219
Chronic pancreatitis—*n* (%)	7 (21)	9 (13)	0.723
FNB Needle *			
22 G Cook Procore—*n* (%)	11 (32)	6 (9)	0.008
20 G Cook Procore—*n* (%)	17 (50)	49 (74)	0.028
25 G Cook Procore—*n* (%)	2 (6)	2 (3)	0.880
22 G Boston Acquire—*n* (%)	4 (12)	6 (9)	0.944
Other—*n* (%)	0 (0.0)	3 (5)	0.520

*: for the patients of the repeat EUS FNB, only the first FNB needle is indicated. EUS: Endoscopic Ultrasound. FNB: Fine Needle Biopsy. G: Gauge.

**Table 2:** Operating characteristics of EUS FNB for solid pancreatic lesions.

	Reapeat EUS FNB *n* = 34	Single EUS FNB *n* = 68	*p* Value
Sensitivity	75%	57%	0.554
Specificity	81%	92%	0.119
Positive predictive value	96%	95%	0.804
Negative predictive value	33%	44%	0.629
Diagnostic accuracy	80%	88%	0.824
Sample adequacy	91%	94%	0.783

EUS: Endoscopic Ultrasound. FNB: Fine Needle Biopsy.

**Table 3:** Diagnostic accuracy of a first and a repeat EUS-guided fine needle biopsy for the diagnosis of solid pancreatic lesions.

	Entire Cohort (*n* = 102)	Repeat EUS FNB (*n* = 34)	Single EUS FNB (*n* = 68)	*p*
Final diagnosis on pathology-*n* (%)	90 (88)	25 (74)	65 (96)	0.05
Pancreatic adenocarcinoma-*n* (%)	71 (70)	20 (60)	51 (75)	0.094
Neuroendocrine tumor-*n* (%)	14 (14)	3 (9)	11 (16)	0.476
Autoimmune pancreatitis-*n* (%)	3 (3)	3 (9)	1 (2)	0.534
Pancreatic metastasis-*n* (%)	2 (2)	1 (3)	1 (2)	1.000
Chronic pancreatitis-*n* (%)	4 (4)	5 (15)	1 (2)	0.207
Other–*n* (%)	7 (7)	2 (6) *	3 (5) **	0.332

*: degenerated IPMN, and normal pancreas. **: degenerated IPMN and two normal pancreas. EUS: Endoscopic Ultrasound. FNB: Fine Needle Biopsy. IPMN: Intraductal Papillary Mucinous Neoplasm.

## Data Availability

Data will be made available to researchers who contact the corresponding authors and provide a methodologically sound proposal.
